# Generating Cloned Goats by Somatic Cell Nuclear Transfer—Molecular Determinants and Application to Transgenics and Biomedicine

**DOI:** 10.3390/ijms22147490

**Published:** 2021-07-13

**Authors:** Maria Skrzyszowska, Marcin Samiec

**Affiliations:** Department of Reproductive Biotechnology and Cryoconservation, National Research Institute of Animal Production, Krakowska 1 Street, 32-083 Balice n. Kraków, Poland

**Keywords:** domestic goat, somatic cell cloning, SCNT-derived embryo, genetically engineered specimen, gene targeting, genome editing, biopharmacy, biomedicine, nutri-biotechnology

## Abstract

The domestic goat (*Capra aegagrus hircus*), a mammalian species with high genetic merit for production of milk and meat, can be a tremendously valuable tool for transgenic research. This research is focused on the production and multiplication of genetically engineered or genome-edited cloned specimens by applying somatic cell nuclear transfer (SCNT), which is a dynamically developing assisted reproductive technology (ART). The efficiency of generating the SCNT-derived embryos, conceptuses, and progeny in goats was found to be determined by a variety of factors controlling the biological, molecular, and epigenetic events. On the one hand, the pivotal objective of our paper was to demonstrate the progress and the state-of-the-art achievements related to the innovative and highly efficient solutions used for the creation of transgenic cloned does and bucks. On the other hand, this review seeks to highlight not only current goals and obstacles but also future challenges to be faced by the approaches applied to propagate genetically modified SCNT-derived goats for the purposes of pharmacology, biomedicine, nutritional biotechnology, the agri-food industry, and modern livestock breeding.

## 1. Introduction

**Figure 1:**
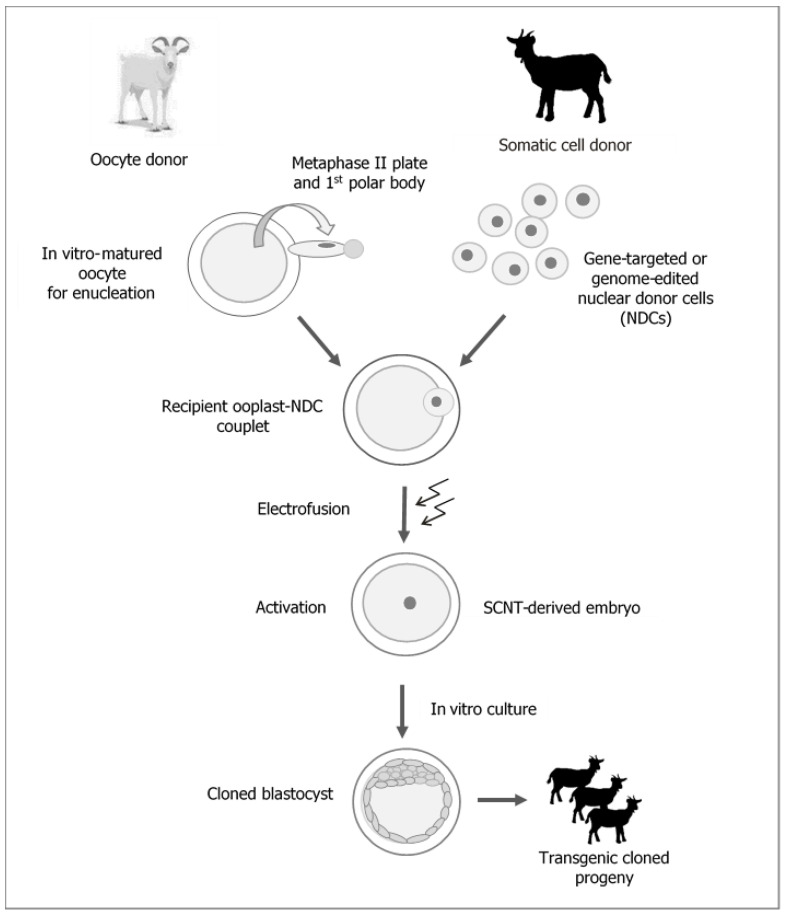
Generation of transgenic cloned goats by somatic cell nuclear transfer (SCNT).

One of the most rapidly developing strategies for reproductive biotechnology in mammals, including farm livestock species, is cloning by somatic cell nuclear transfer (SCNT) (Figure 1).

It is beyond any doubt that the attractiveness of cloning techniques results from their potential to generate and multiply transgenic animals, which are valuable due to the expression of modified genes (Figure 1). Furthermore, this attractiveness also depends, to a lesser degree, on the possibility to replicate individuals with excellent, highly heritable breeding (genetic) and performance traits, which may shorten the generation interval and increase the rate of breeding progress. However, the aforementioned areas of research are being explored on a limited scale due to the high costs associated with the cloning procedure resulting from the low efficiency of the method. It is beyond any doubt that the widespread use of cloning methods will be possible once efficacy and repeatable results are guaranteed [1,2,3,4].

The main reason for low pre- and postimplantation developmental potential and poor quality of SCNT-derived embryos is the abnormal adaptation of the transferred somatic cell nuclei to the biochemical conditions of the oocyte cytoplasmic microenvironment, i.e., their incomplete or improper remodeling and reprogramming in the cytoplasm of nuclear-transferred oocytes. The latter also gives rise to the relatively high incidence of congenital malformations (anatomo-, histo-, and physiopathological changes) in cloned fetuses and offspring. This calls for studies aimed at the precise determination of the conditions that facilitate epigenetic reprogramming in the nuclear donor cell genome during the pre- and postimplantation development of SCNT-generated embryos and fetuses of different mammalian species, including the domestic goat [5,6,7,8,9,10]. Promising results were achieved by investigations that focused on the use of extrinsic nonselective agents for stimulating the epigenetically regulated transcriptional activity of genomic DNA in both nuclear donor somatic cells and SCNT-cloned embryos [11,12,13,14,15].

## 2. Key Issues Related to Biological, Molecular, and Epigenetic Determinants Affecting the Efficacy of Somatic Cell Cloning in Goats

The provenance of somatic cells is a factor that can have a significant impact on cloning efficiency in goats. Relatively few types of nuclear donor cells have been tested for their suitability for the production of cloned embryos, fetuses, and/or offspring in this livestock species. Those that have been tested include cells stemming from several types of tissues collected from both caprine fetuses and adult animals of both sexes and of different ages. Among the nuclear donor cells (NDCs) used for SCNT procedures, mention should be made of: (1) in vitro cultured (transgenic or nontransgenic) fetal dermal fibroblasts [12,16,17,18,19,20,21,22]; (2) juvenile and adult dermal fibroblasts [14,23,24,25,26]; (3) mural granulosa cells isolated from antral ovarian follicles [18,27]; (4) cumulus oophorus cells [18,27,28]. 

Special consideration should be given to the use of pituicytes, i.e., endocrine cells originating from the anterior pituitary (also known as adenohypophysis) of postpubertal bucks, as a source of nuclear donors for SCNT in goats [29]. To date, this cerebral tissue-specific type of endocrine cell, which synthesizes and secretes tropic hormones, has not been used for SCNT in other species of farm and laboratory animals. It has been postulated, however, that artificial (ectopic) control of metabolic and secretory activities of the endocrine compartment in all lobes of the pituitary gland of livestock species might be feasible through genetic modification (transfection) of pituicytes at the in vitro culture level. In turn, utilizing genetically transformed pituitary-derived glandular cells, which are characterized by inducible expression of recombinant human hormonal proteins or polypeptides, for the generation of transgenic specimens of different mammalian species by somatic cell cloning opens up a variety of new application opportunities. The latter encompass the production of transgenic animal bioreactors, which provide xenogeneic (human) tropic hormones in cytosol extracts (homogenates) of pituicytes or in blood plasma. These hormones are indispensable for the clinical application of therapies for many human monogenic diseases, which induce endocrine-mediated congenital malformations. The transfer of caprine SCNT embryos that had been reconstructed with pituicytes into the reproductive tract of hormonally synchronized recipient females resulted in the birth of a cloned male kid. The results of these experiments confirmed that even the nuclear genome of terminally differentiated somatic cells such as pituicytes can successfully undergo the complete processes of epigenetic remodeling and reprogramming in pre- and postimplantation cloned goat embryos [29].

Deng et al. [30] examined the methylation profile and expression level of the *Xist* (X-inactive specific transcript) gene in the cells of SCNT embryos and in ear fibroblast cells, lung-derived cells, and cerebral cells collected from deceased cloned goats. The methylation profile observed for the *Xist* gene, which is transcribed into a noncoding mRNA molecule, i.e., a transcript that does not exhibit translational activity, was higher in 8-blastomere-stage SCNT embryos as compared to their in vitro-fertilized embryo counterparts generated by intracytoplasmic sperm injection (ICSI). Moreover, an increased methylation profile of the *Xist* gene was observed in the cells stemming from explants representing tissues/organs such as conchal skin, lungs, and brain, isolated postmortem from dead 3-day-old cloned female kids in relation to naturally bred specimens. While for ear skin-derived tissue bioptates originating from live cloned does, and for lung and brain tissue samples retrieved from dead cloned kids, the methylation profile of 5′-cytidine-3′-monophopshate-5′-guanosine-3′ (CpG) islands within the differentially methylated regions/imprinting control regions (DMRs/ICRs) of the *Xist* gene remained unchanged. Therefore, the transcriptional activity of the *Xist* gene diminished remarkably in the lungs and brain of dead cloned does, resulting in a lack of inactivation recognized for one of the X chromosomes (either of paternal or of maternal origin) in the cells of the previously indicated organs. In turn, a significant increase in *Xist* gene expression was shown in the ear-derived cutaneous fibroblast cells of live cloned does. This contributed to the normal inactivation of one of the two X chromosomes in these specimens. It is evident from this study that an increased incidence of hypermethylation and transcriptional suppression of the *Xist* gene, and thus no inactivation of one of the two X chromosomes, or in other words, active initiation of enhanced transcriptional activity (i.e., biallelic overexpression) of the genes localized in the loci of the paternal and maternal X chromosomes occurred in caprine SCNT-derived female fetuses. For these reasons, the aforementioned processes, which were also identified in the tissue explants recovered from 3-day-old dead cloned does, are found to arise from incomplete and aberrant reprogramming and were highlighted as a result of the epigenetically determined transcriptional activity of the somatic cell nuclear genome in cloned goat embryos [30]. 

Incomplete or incorrect epigenetic reprogramming of epigenetic memory, which is encoded in extragenic covalent modifications of the somatic cell nuclear genome, was found to be one of the main factors decreasing the efficiency of somatic cell cloning in mammals, including the domestic goat. Reductions in this efficiency are reflected in the weakened in vitro and/or in vivo developmental potential of SCNT-derived embryos [7,8,31]. Methylation of cytosine residues in CpG islands/dinucleotides is a widely explored/recognized modification of the somatic cell nuclear genome in cloned embryos [6,9,10,32]. Han et al. [33] demonstrated that enzymatic activity of ten-eleven translocation methylcytosine dioxygenase 3 (TET3) is a key molecular mechanism underlying active DNA demethylation in preimplantation goat embryos created by somatic cell cloning. Knocking out the *TET3* gene led to the inhibition of active (i.e., DNA replication-independent) demethylation of 5-methylcytosine (5-mC) residues in 2-blastomere-stage cloned goat embryos. As a consequence, this brought about the downregulation of the expression of the pluripotency-related *Nanog* gene in the inner cell mass (ICM) compartment of the generated blastocysts. In turn, overexpression of the *TET3* gene that had been induced by transgenization of in vitro cultured somatic cells resulted in: (1) abundant demethylation of DNA 5-mC residues; (2) declined quantitative profile of 5-mC moieties; (3) increased incidence of 5-hydroxymethylcytosine residues; (4) intensified transcriptional activity of crucial pluripotency-related genes. Furthermore, the use of genetically transformed somatic cells displaying overexpression of the *TET3* gene—as nuclear donors for the reconstruction of caprine enucleated oocytes—contributed to an enhancement in the extent of active demethylation of 5-mC residues within somatic cell-inherited nuclear DNA. The latter perpetuated hypomethylation of the somatic cell-derived genome in cleaved SCNT embryos, subsequently triggering remarkable improvements in their in vitro and in vivo developmental capabilities. It follows that overexpression of the *TET3* gene in NDCs significantly ameliorates the efficacy of somatic cell cloning in goats.

The developmental potential of the mammalian SCNT embryos, including their caprine representatives, which inherit the somatic cell nuclear genome as a result of the reconstruction of enucleated oocytes, is highly dependent on the level of epigenetic modifications within DNA and chromatin-derived histones of the NDCs undergoing long-term in vitro culture [34,35,36]. One of the strategies to reverse advanced alterations in the pattern of epigenetic covalent modifications within somatic cell nuclei, which encompass rapid DNA methylation and a decrease in the quantitative profile of histone protein acetylation, appears to be the exposure of NDCs, SCNT-derived oocytes, and corresponding embryos to reversible agents inhibiting biocatalytic activity of DNA methyltransferases (DNMTs) and/or histone deacetylases (HDACs). The use of nonselective or selective promoters of epigenetically determined transcriptional activity of genomic DNA in both in vitro cultured NDCs and cloned embryos is supposed to be an approach that allows for proper reprogramming of somatic cell nuclei [7,12,14]. Exogenously modulating the epigenetic memory profile of genomic DNA appears to contribute to successfully reversing the “transcriptional clock” of a differentiated somatic cell nucleus to the status of a cell nucleus characteristic of a totipotent or pluripotent embryonic cell. As a consequence, such efforts induce the restoration of the expression pattern that is seen in genes that are inevitable in the initiation and progress of the developmental program of SCNT-derived embryos [37,38]. This results in a reduction in the methylation degree of DNA cytosine residues and an increase in the acetylation of nuclear chromatin histone proteins [39,40]. In turn, the previously specified processes were shown to bring about recapitulation and perpetuation of the correct and faithful profiles of transcriptional activities observed both for the genes indispensable to induce and maintain the totipotency/pluripotency states and for the genes encoding enzymes responsible for endogenous epigenetic modifications during pre- and postimplantation embryogenesis. The totipotency/pluripotency-related genes encompass those that encode such proteins as: e.g., octamer-binding transcription factor 3/4 (Oct3/4), the homeobox-containing transcription factor Nanog, whose name stems from the Celtic/Irish mythical word Tír na nÓg (i.e., The Land of the Ever-Young), DNA-binding proto-oncogenic/oncogenic transcription factor c-Myc, sex-determining region Y (SRY)-box 2 transcription factor (Sox2), Krüppel-like factor 4 (Klf4), reduced expression protein 1 (Rex1), and caudal-type homeobox protein 2 (Cdx2). The epigenetic modifier genes involve those that encode such enzymatic proteins as: e.g., DNA methyltransferase type 1 (DNMT1), DNA methyltransferase type 3a (DNMT3a), DNA methyltransferase type 3b (DNMT3b), histone deacetylase type 1 (HDAC1), histone deacetylase type 2 (HDAC2), histone methyltransferase (HMT), and histone acetyltransferase (HAT) [3,41,42]. In the wake of recent research, innovative and highly efficient methods were developed to modulate the epigenetic memory profile of mammalian SCNT embryos, including their caprine counterparts. These methods are focused on applying exogenous nonselective HDAC inhibitors (such as trichostatin A, valproic acid, and scriptaid) and/or nonselective DNMT inhibitors (such as 5-aza-2′-deoxycytidine) or selective inhibitors lysine K4 demethylases specific for histones H3 within the nucleosomal core of nuclear chromatin (such as trans-2-phenylcyclopropylamine (tranylcypromine; 2-PCPA)). The aforementioned strategies may considerably modify the epigenetically determined reprogramming of the somatic cell nuclear genome in SCNT-derived embryos. The final results of these innovative solutions turn out to be significant enhancements of the pre- and/or postimplantation developmental competence and an improvement in the molecular quality of cloned embryos in mammals, including the domestic goat [12,13,14,42,43,44]. 

## 3. Species-Specific Advantages of the Goat That Increase the Potential for Its Practical Application in Transgenics, Biopharmacy, Biomedicine, and Biotechnology

The domestic goat (*Capra aegagrus hircus*), a species with a tremendously high biodiversity of breeds showing relatively high milk and/or meat yield, may serve as an excellent research subject for SCNT-mediated production of transgenic bioreactor specimens. These caprine genetically engineered bioreactors of foreign species-descended (xenogeneic) bio-preparations can provide recombinant human therapeutic proteins that are designated as biopharmaceuticals or nutraceuticals (Table 1), together with physiological secretions (e.g., milk) and excreta (e.g., urine). Moreover, somatic cell cloning in this livestock species seems to be a reliable, feasible, and powerful tool for generating and/or multiplying specimens (does and bucks) that display genetically modified parameters of meatiness and intramuscular adipose tissue content (Table 1). Increasing the efficiency of producing purified xenogeneic biopharmaceuticals or bionutraceuticals derived from the mammary glands (udders) of transgenic goats would thus allow them to be phased into the biopharmaceutical industry [24,45]. Another tangible benefit of producing transgenic cloned goats, which appears to be especially valuable for the xenogeneic product of the transgenic expression of exogenous DNA (directed at the mammary glands or resulting in higher meatiness), is the relatively short species-specific generation interval. The latter allows for increasing the rate of genetic progress in the breeding of founder does and bucks [24,25]. Yet another advantage of this small ruminant species is the low susceptibility of dairy and meat goats to infection with pathological prions (PrP^Sc^) that cause scrapie in sheep [27,46,47]. Transgenic goats may serve as optimal bioreactors to produce human therapeutic proteins for various agroeconomic reasons. Compared to the breeding of transgenic cows, these animals are more easily farmed, their natural and biotechnologically assisted reproduction can be more rapidly controlled, and they are much cheaper to keep as compared to large ruminants. Relative to their body size, they have fairly large udders with a predominance of glandular tissue over fibrous parenchyma, which makes this small ruminant species genetically predisposed to a high production potential of colostrum and milk. The possible consequence of these anatomo-physiological advantages in goats is the high performance of transgenic doe herds in terms of lactogenic synthesis and secretion of recombinant human therapeutic proteins (biopharmaceuticals or nutraceuticals) by alveolar mammary epithelial cells. These caprine cells provide udder-derived secretion with a genetically modified qualitative and quantitative composition [24,48,49]. 

The first transgenic cloned kids were generated from the nuclear-transferred embryos reconstructed with caprine somatic cells that had been previously transfected in vitro with relatively simple gene constructs. These gene constructs contained no genomic sequences of the regions encoding structural transgenes, but were composed of the exon segments of genes encoding selectable marker proteins, e.g., *PGKneo* fusion genes. The aforementioned fusion genes are comprised of murine phosphoglycerate kinase (*PGK*) promoters and neomycin phosphotransferase (*neo*) genes. The *neo* gene determines resistance to selective aminoglycoside antibiotic designated as geneticin disulphate (G418 sulphate). In the study by Zou et al. [19], in vitro cultured fetal fibroblasts provided a source of transgenic NDCs for SCNT-based cloning in goats. The transgenic NDCs were created by their transfection with a gene construct that only contained the *neo* gene. The above-mentioned study resulted in the production of five genetically modified kids. In turn, Keefer et al. [17] and Baldassarre et al. [27,46] used the in vitro lipofection approach to transfect fetal fibroblasts with a more complex plasmid gene construct (*CEeGFP*). The *CEeGFP*-fusion gene was composed of: (1) the enhanced green fluorescent protein (*eGFP*)-reporter gene driven by the human elongation factor-1α promoter and cytomegalovirus enhancer and (2) the *neo* gene under the control of the simian virus-40 (*SV-40*) promoter. Following the transfer of genetically transformed cloned embryos into the reproductive tracts of recipient surrogates, one cloned doe showing the expression of the *eGFP*-reporter transgene was produced.

Transgenic animals with the high transcriptional activity profiles (diagnosed in vivo) of a xenogeneic gene may be subsequently multiplied by somatic cell cloning. This is particularly justified when biopharmaceuticals stemming from these animals may find widespread application in the treatment of patients suffering from various single-gene heritable disorders. When transgenic biopharmaceuticals obtain certification for application in humans, somatic cell cloning of genetically modified specimens will allow, at least in theory, for the maintenance of homogeneity of the drugs extracted from natural secretions and excretions (milk, urine) of the successive generations of cloned animals. This technology was successfully used by the American biotechnology company GTC Biotherapeutics (formerly Genzyme Transgenics Corporation), which generated transgenic goats exhibiting monoallelic expression of recombinant human antithrombin III gene (*rhAT*) in their mammary glands (udders). The production of transgenic cloned goats was based on the use of a standard intrapronuclear microinjection into the zygotes of cDNA constructs containing the goat β-casein gene promoter. In the performed experiments, genetically modified fibroblast cell lines were established from fetuses obtained by mating nontransgenic does with a genetically modified founder buck. This buck displayed transcriptional activity of the *rhAT* gene that was directed into the mammary gland (udder). Clonal lines of transgenic fetal fibroblast cells served as a source of nuclear donors in the somatic cell cloning procedure, which resulted in a total of eight genetically engineered SCNT-derived female kids [16,45]. The findings of Cheng et al. [28] represent another example of applying the somatic cell cloning technique for multiplying populations of genetically transformed specimens. In this case, enucleated oocytes were reconstructed by SCNT with the use of in vitro cultured fibroblast cell lines collected from dermal tissue explants of a transgenic goat displaying ubiquitous expression of recombinant human erythropoietin (rhEPO). After surgical transfer of the cloned embryos into the reproductive tracts of hormonally synchronized recipient does, two genetically modified kids were born. The SCNT-derived offspring were characterized by mammary gland-specific expression of xenogeneic rhEPO protein.

## 4. Transgenic Cloned Goats as Bioreactors That Produce Recombinant Human Therapeutic Proteins

The nuclear transfer of in vitro-transfected somatic cells increases the probability of producing nonmosaic transgenic offspring, which have an exogenous gene construct incorporated into the primordial germ cell line. Such specimens, which are identified as nonchimeric with regard to the genetic transformation of gametogenic and somatic cells, retain their full capacity to transmit phenotypically and molecularly diagnosed transgene expression to the secretory epithelial cells (lactocytes) in the mammary glands of the next generation of kids [49,55,56]. An outstanding example is found in the findings of Baguisi et al. [45]. High-level expression of the *rhAT* gene detected in the udder lactogenic cells of three cloned does, which were produced from SCNT embryos reconstructed with transgenic fetal fibroblasts, was also reflected in the very high phenotypic value of this genetically modified trait in the milk samples. Over a 33-day lactation induced at 2 months of age, the milk yield of these genetically engineered does reached approximately 160 mL. Additionally, the rhAT concentration in the collected milk was maintained at a level of as much as 5.8 g/L (20.5 U/mL was observed for the enzymatic activity of purified biopharmaceutical) at day 5 and 3.7 g/L (14.6 U/mL for the biocatalytic activity) by day 9 of lactation. At such high concentrations of recombinant therapeutic proteins in milk, large-sized herds of transgenic goats could easily yield 300 kg of extracted (purified) biopharmaceutical product per year. Combining somatic cell cloning technology with hormonal induction of early lactation in prepubertal transgenic does will shorten the time needed to obtain the transgene expression product by as much as 8 to 9 months from the time of cell line transfection to the secretion of the genetically engineered protein biopreparation into milk [20,45]. On the one hand, the volume of these retrieved milk samples is sufficient for estimating the recombinant protein yield. On the other hand, taking into account even a relatively low quantitative profile of translational activity identified for transgene-transcribed mRNA (as measured by milligram quantities of therapeutic protein per 1 mL of milk), this amount of milk can be used for multiple clinical tests of the pharmacokinetic, hormonal, and enzymatic activity of the produced biopharmaceuticals.

Special consideration should be given to the broad international commercialization of the first biopharmaceutical in 2006–2009, designated as ATryn^®^, by GTC Biotherapeutics. The basic active biochemical component of this pharmacological biopreparation is rhAT, which was recovered from the milk synthesized and secreted by the udders of transgenic cloned specimens of a large livestock species, the domestic goat [47,57,58]. This is a milestone in the practical, commercial-scale implementation of the first biopharmaceutical product of modern mammalian reproductive biotechnology based on embryonic genome engineering technologies such as transgenesis and somatic cell cloning of farm animals. It is worth pointing out here that ATryn^®^ is the world’s first drug to be provided by mammary gland-based bioreactors of genetically engineered cloned goats that exhibit highly efficient and organ-specific mono- or biallelic expression of the *rhAT* transgene. This biopharmaceutical was originally granted a marketing authorization by the European Medicines Agency (EMA) in 2006 for use in the biopharmaceutical and medical sector of the European Union, followed by certification from the United States Food and Drug Administration (FDA or USFDA) in 2009 for marketing in the biopharmaceutical and biomedical sector in the USA and Canada [1,59]. At this stage, ATryn^®^ is widely used in biomedical programs/therapeutic platforms for the treatment of hereditary AT deficiency in hospitalized medical patients [60,61].

Another example of the practical application of the mammary glands of genetically transformed cloned goats as bioreactors to synthesize human therapeutic proteins or so-called humanized milk is found in the study by Zhu et al. [21]. This investigation was aimed at ameliorating allergic reactions and inflammatory responses to β-lactoglobulin (BLG) protein (Table 1). As a major whey protein with potential allergenic effect, BLG occurs in the milk of all even-toed mammals (*Artiodactyla*), including the domestic goat. It has no allergenic properties in human milk. The presence of this protein in caprine milk considerably limits, to a high degree, the consumption of this lactogenesis-derived product despite its high nutritive value and health-promoting benefits. Using conventional homologous recombination, the above-mentioned investigators were the first to functionally inactivate a single copy of the *BLG* gene, either through *BLG* gene knockout or through *hLA* (human α-lactalbumin) gene knock-in into the nuclear genome of in vitro cultured fetal fibroblast cells. These cells subsequently provided a source of nuclear donors for reconstructing enucleated doe oocytes in the somatic cell cloning procedure. The ultimate outcome of this research was the birth of three SCNT-derived kids, among which mono-allelic knockout of the targeted *BLG* gene was confirmed in two specimens (Table 1) [21].

In turn, Yuan et al. [22] used the strategy of somatic cell cloning to generate transgenic goats whose udders were bioreactors that synthesized humanized milk containing pharmaceutical or nutraceutical immune glycoprotein, known as recombinant human lactoferrin (hLF). Genetically transformed fetal fibroblasts provided the source of nuclear donor cells for the reconstruction of enucleated oocytes by somatic cell cloning. The genome of nuclear donor cells had been previously edited by inserting *hLF* cDNA into the *BLG* locus, i.e., by replacing the *BLG* gene with the *hLF* gene. Editing the nuclear genome of fetal fibroblast cells had been mediated by transcription activator-like effector nucleases (TALENs). The efficiency of the targeted mutagenesis observed for the *BLG* gene oscillated at the level of approximately 10%. The results of investigations by Yuan et al. [22] confirmed that the combination of TALEN-based genome editing with the SCNT strategy gave rise to biallelic inactivation of the *BLG* gene through the knock-in of *hLF* exons into the genomic DNA of cloned goats whose mammary glands were targeted by programmed genetic transformation to produce recombinant hLF (Table 1). Transgenically encoded qualitative and quantitative modification of the biochemical composition of caprine milk that subsequently brought about the production of humanized milk in the udders of SCNT-derived goats appears to ameliorate its allergenicity. Diminishing the capability of the milk provided by genetically engineered bioreactors to trigger acute allergic reactions in humans is simultaneously reflected in enriching the goat milk with the valuable multipotent protein designated as LF. This immune glycoprotein, apart from physiologically regulating the dynamic homeostasis of the metabolism of iron cations, is characterized by several other desirable immunotherapeutic properties, including antimicrobial (antibacterial, mycostatic, and antiviral), immunomodulatory, anti-inflammatory, and anticancer abilities (Table 1) [22,24,56].

It is also noteworthy that Zhang et al. [50] reported the effective integration of the recombinant *hLF* gene with the xenogeneic host genome as a result of genetically transforming the caprine fetal fibroblast cells under in vitro culture conditions. The genetically transformed fetal fibroblasts were subsequently used to generate transgenic kids (does) with the *hLF* gene in ear skin tissue samples by somatic cell cloning (Table 1). Out of the six transgenic cloned kids produced, three does died during the perinatal period due to severe bronchopulmonary dysplasia in underdeveloped lungs and acute hypoxemic respiratory failure. The epigenetic analysis of tissue explants collected postmortem from the lungs of perished transgenic does revealed hypermethylation of CpG islands/dinucleotides within the DMR/ICR domain of the gene encoding insulin-like growth factor 2 receptor (IGF2R). For that reason, the maternal allele of the *IGF2R* gene was found to be transcriptionally overactive/upregulated due to enhanced methylation of cytosine residues in the DMR/ICR-associated intron sequence, while its paternal counterpart was shown to be transcriptionally silenced due to the occurrence of parent-of-origin and allele-specific methylation imprint. As a result, the overexpression of mRNA transcribed by the maternal allele of the *IGF2R* gene that had undergone aberrant genomic imprinting was identified in the cell samples of postmortem isolated lung tissue explants stemming from the cloned kids [50]. 

In summary, imprinted genes are an important epigenomic regulator of anatomo-histological growth and development and physiological maturation of the lungs. In turn, aberrant or incomplete reprogramming of the epigenetically determined transcriptional activity of DNA, which underlies abnormal (i.e., increased) methylation of cytosine moieties within DMR/ICR-related intron sequences of the imprinted maternal allele of the *IGF2R* gene, determines the monoallelic overexpression of this gene exprimed from the maternal genome in the lungs of cloned fetuses. The latter appears to be one of the main lethal factors positively correlated with etiopathogenesis of lung hypoplasia and acute pulmonary insufficiency in neonatal transgenic cloned kids [50].

## 5. Transgenic Cloned Goats as a Source of Valuable Meat for Humans

Attempts to create and multiply transgenic cloned goats may provide a research basis for the SCNT-mediated generation of genetically engineered specimens (bucks and does) that exhibit genotypic and phenotypic modifications related to increased carcass meatiness and decreased intramuscular fatness. These animals could serve as a valuable research model for expanding our knowledge of the importance of myostatin, e.g., in the context of the quality and taste of the meat from individuals displaying superior gains in muscle tissue and myofiber size. Myostatin, encoded by the *MSTN* gene, is a hormonal inhibitory polypeptide that inhibits/downregulates the growth, differentiation, maturation, and development of skeletal muscles in mammals. Research shows that functional inactivation of the *MSTN* gene using gene targeting (targeted mutagenesis) or genome editing techniques contributes to increase skeletal muscle mass while diminishing the content of intramuscular adipose tissue and reducing genetically determined or diet-induced obesity. This has the beneficial effects of increased meat yield, fattening performance, and dressing percentage in genetically modified males and females generated by SCNT-based cloning. These effects are evoked by cellular hyperplasia (proliferation) and hypertrophy (enlargement) of striated muscle tissue (Table 1). The hyperplasia and hypertrophy of striated muscle cells (i.e., skeletal sarcocytes known as syncytial myocytes) are synergistically triggered as a result of the following:

– Expediting differentiation of the predominant multipotent muscle stem cells (i.e., satellite cells) and their myogenic progenitor cell derivatives into primary myoblasts;
– Accelerating the proliferative growth of mononucleated myoblasts;
– Facilitating cyto- and histophysiological maturation of genetically engineered skeletal muscle tissue by syncytial fusion of myoblasts and their conversion (transformation) into myotubes and the resultant multinucleated myofibers;
– Enlargement of muscle fibers;
– Extension of myofiber lengths and individual sarcomere lengths in whole muscle fibers;
– Increase in both myofibrillar volume and myofiber number [25,62,63].

It is beyond any doubt that SCNT-derived goats that are characterized by *MSTN* gene silencing (Table 1) [25] represent a powerful, reliable, and feasible tool for investigations targeted at nutritional physiology, food technology, dietetics, nutrigenomics, nutriepigenomics, nutritranscriptomics, nutriproteomics, and human nutrition metabolomics and metabonomics. Myostatin gene knockout (*MSTN*-KO) in goats (Table 1) [25,62,64] and sheep [63,65] was investigated in several research centers. However, as a result of the low efficacy of homologous recombination (HR)-mediated targeted mutagenesis, short-hairpin RNA-mediated gene targeting, and zinc-finger nuclease (ZFN)-mediated genome editing, only a few studies resulted in successful *MSTN* gene knockdown in ex vivo expanded caprine juvenile cutaneous fibroblasts [25], ovine fetal myoblasts [63], or ovine fetal cutaneous fibroblasts [65]. 

In recent years, other noteworthy solutions have been applied to genome editing and have been subsequently adapted to ARTs. These were then applied in combination to cloning goats using SCNT. These solutions are aimed at strategies based on the use of TALENs or the clustered regularly interspaced short palindromic repeat/CRISPR-associated endonuclease type 9 (CRISPR/Cas9)-assisted system. The progress achieved in developing and optimizing the techniques of targeted knockout of specific gene loci is promising. Therefore, these techniques are increasingly utilized for precise genome editing, allowing for the genome of mammals, including the domestic goat, to be modified with relative ease [66,67,68,69,70,71]. Ni et al. [53] were the first to demonstrate that CRISPR/Cas9-mediated genome editing can induce accurate mono- or biallelic mutations in the *MSTN* gene of caprine fetal fibroblast cells. The clonal lines of these NDCs that exhibit biallelic *MSTN*-KO were used in a SCNT procedure, resulting in three live-born cloned kids, all of which carried a biallelic mutation in the form of double inactivation of the *MSTN* gene’s loci (Table 1). 

In turn, Yu et al. [54] showed that TALEN-based genetic transformation leads to the successful inhibition of *MSTN* gene expression in gene-edited cloned goats (Table 1). Moreover, the outcome of both TALEN- and CRISPR/Cas9-mediated systems that were applied to edit the nuclear genome of SCNT-derived Alpas breed cashmere goats (Table 1) was evaluated by Zhang et al. [52]. The efficiency of triggering *MSTN*-KO was compared at many levels of pre- and postimplantation development of transgenic cloned embryos. The rates of both electro-transfecting/electroporating the somatic cells and cutting exon 1 within the *MSTN* gene were found to be higher for the CRISPR/Cas9-assisted strategy of genome editing than for its TALEN-mediated counterpart. Nevertheless, the genome-wide off-target effects were shown to be more frequent for the CRISPR/Cas9-mediated system than for the TALEN-mediated system. Furthermore, for CRISPR/Cas9-based genome editing, the incidence of effectively inducing targeted biallelic mutagenesis of the *MSTN* gene increased over eight times as compared to TALEN-based genome editing. In turn, caprine SCNT embryos that had been reconstructed with TALEN-mediated transgenic NDCs reached the 8-blastomere stage more quickly and their cleavage activity was significantly higher as compared to SCNT embryos derived from CRISPR/Cas9-mediated gene-edited NDCs. However, cloned kids were produced, following the surgical transfer of SCNT embryos into recipient does, that stemmed from NDCs that were genetically modified only using the CRISPR/Cas9-assisted technique (Table 1). This ultimately suggests that the high yield of generating targeted modifications of the *MSTN* gene (*MSTN*-KO) was achieved using CRISPR/Cas9-mediated genome editing [52].

To summarize, the study by Zhang et al. [52] proved that, although the TALEN-dependent genome transformation strategy has a certain advantage over the CRISPR/Cas9-dependent system, the latter offers significant benefits related to the precision programmed editing of the genes (Table 1). This makes this system a powerful and high-performance genetic engineering tool for livestock breeding practice and, in particular, in the fields of agri-food biotechnology and human food technology (nutritechnology) based on a meat diet [52,68,69,70,71].

## 6. Conclusions and Future Goals

Although the efficiency of somatic cell cloning in goats remains relatively low, further studies are necessary because modern ART has important implications in the fields of goat breeding, the transgenics of this mammalian species, agri-food biotechnology, biomedicine, and biopharmacy.

An increase in the efficiency of somatic cell cloning techniques in the domestic goat can be brought about by further intensive research into improving both developmental competence and the parameters related to the molecular and epigenetic quality of SCNT-derived embryos. The latter can be achieved by efforts aimed at using nonselective or selective inhibitors of DNMTs and HDACs, which would in turn lead to enhancements in the reprogrammability of the epigenetic memory profile within genomic DNA of NDCs, nuclear-transferred oocytes, and the corresponding caprine cloned embryos. This is a sine qua non condition for the practical use of SCNT-based cloning, and thus for the production of genetically transformed goats for the purposes of human nutrition technology based on a meat diet. The main focus of the aforementioned efforts is the successful SCNT-mediated creation and multiplication of transgenic does and bucks with enhanced meat yields due to cellular hyperplasia and hypertrophy within skeletal muscle tissue. This is also a basic requirement for the effective propagation of genetically engineered or genome-edited does for the biopharmaceutical and nutraceutical industry. An ideal example of this is the generation of transgenic goats whose udders serve as bioreactors for recombinant human therapeutic proteins or biochemically humanized milk.

## Figures and Tables

**Table 1:** Targets and effects of genetic modification in transgenic cloned goats.

Target of Genetic Modification	Strategy of Genetic Modification	Method of Somatic Cell Transfection	Genotypic Effect of Genetic Modification	Phenotypic Effect of Genetic Modification	Reference
Mammary gland (udder)	Gene targeting (HR-mediated targeted mutagenesis)	Electroporation	- HR-induced disruption of caprine the *BLG* gene by: either its monoallelic knockout or knock-in of the *hLA* gene construct into the *BLG* exon	- Functionally inactivating the caprine *BLG* gene - Targeted expression of the *hLA* gene in the lactogenic cells of udder-based bioreactors - Synthesis of upgraded or humanized milk without allergenic (BLG-triggered) properties	[21]
		Lipofection	- HR-dependent targeted incorporation of *hLF* cDNA into the nuclear genome (under the control of the goat β-casein gene promoter)	- Mammary gland-specific monoallelic expression of the *hLF* gene - Udder-mediated synthesis of upgraded or humanized milk - Production of genetically engineered milk (GEM) characterized by a broad spectrum of hLF-induced immunotherapeutic properties - GEM properties determined by antimicrobial, immunomodulatory, anti-inflammatory, and anticancer attributes of hLF	[50,51]
	TALEN-mediated gene editing	Electroporation	- TALEN-dependent targeted insertion of *hLF* cDNA at *BLG* locus resulting in: biallelic knock-in of the *hLF* coding sequence into the *BLG* exon	-Targeted expression of the *hLF* gene in udder-based bioreactors - Synthesis of hLF-enriched or humanized milk - Production of GEM displaying immunotherapeutic properties	[22]
Skeletal muscles	Gene targeting (HR-mediated targeted mutagenesis)	Lipofection	- Monoallelic knockout (semi-deficiency) of the *MSTN* gene in SCNT-derived progeny	- Inducing hyperplasia and hypertrophy of striated muscle cells -Remarkably gaining skeletal muscle mass and augmenting meatiness by genetically transforming the muscular system of heterozygous (*MSTN^+/−^*) transgenic cloned offspring	[25]
	CRISPR/Cas9-mediated gene editing	Electroporation	- Monoallelic knockout (semi-deficiency) of the *MSTN* gene in SCNT-derived progeny	- Expression of cellular hyperplasia and hypertrophy in genome-edited (GE) skeletal muscle tissue of heterozygous (*MSTN^+/−^*) transgenic cloned offspring	[52]
		Nucleofection	- Biallelic knockout (deficiency) of the *MSTN* gene in SCNT-derived progeny	- Expression of myofiber hyperplasia and hypertrophy in GE muscular system of homozygous (*MSTN^−/−^*) transgenic cloned offspring	[53]
	TALEN-mediated gene editing	Electroporation	- Monoallelically knocking out the *MSTN* gene - Biallelically knocking out the *MSTN* gene in SCNT-derived progeny	- Triggering hyperplasia and hypertrophy of skeletal myocytes in the GE muscular system of: either heterozygous (*MSTN^+/−^*) transgenic cloned offspring or their homozygous (*MSTN^−/−^*) counterparts	[54]
		Electroporation	- Monoallelic knockout of the *MSTN* gene (*MSTN^+/−^*) in NDCs - Biallelic knockout of the *MSTN* gene (*MSTN^−/−^*) in NDCs	- Onset of the mono- or biallelically transcriptionally silencing *MSTN* gene in isozygous GE NDCs - Failure in the generation of GE cloned progeny exhibiting phenotypes determined by *MSTN* mono- or biallelic deletion	[52]

## Data Availability

Not applicable.

## Abbreviations

ART	Assisted reproductive technology
BLG	β-lactoglobulin
Cas9	CRISPR-associated endonuclease type 9
Cdx2	A product transcribed from proto-oncogene/oncogene encoding caudal-type homeobox protein 2 that represents the family of intestinal epithelium/adenocarcinoma-specific and DNA-binding homeodomain transcription factors inevitable in intestinal organogenesis
c-Myc	Avian myelocytomatosis viral oncogene homolog encoding a DNA-binding
	proto-oncogenic/oncogenic transcription factor
CpG	5′-Cytidine-3′-monophopshate-5′-guanosine-3′
CRISPR	Clustered regularly interspaced short palindromic repeat
DMR	Differentially methylated region
DNMT	DNA methyltransferase
eGFP	Enhanced green fluorescent protein
GE	Genome-edited
GEM	Genetically engineered milk
HAT	Histone acetyltransferase
HDAC	Histone deacetylase
HMT	Histone methyltransferase
hLA	Human α-lactalbumin
hLF	Human lactoferrin
HR	Homologous recombination
ICM	Inner cell mass
ICR	Imprinting control region
ICSI	Intracytoplasmic sperm injection
IGF2R	Insulin-like growth factor 2 receptor
Klf4	Krüppel-like factor 4 (also called gut-enriched Krüppel-like factor or GKLF); an evolutionarily conserved zinc finger-containing transcription factor that regulates diverse cellular processes such as cell growth, proliferation, differentiation, apoptosis, and somatic cell reprogramming
5-mC	5-Methylcytosine
MSTN	Myostatin
MSTN-KO	Myostatin gene knockout
Nanog	Homeobox-containing transcription factor whose name stems from the Celtic/Irish
	mythical word Tír na nÓg (i.e., Tir Na Nog; The Land of the Ever-Young)
NDC	Nuclear donor cell
Oct3/4	Octamer-binding transcription factor 3/4 (also designated as POU5F1); a member of the family of POU (Pit-Oct-Unc)-domain and homeodomain transcription factors
2-PCPA	Trans-2-phenylcyclopropylamine; Tranylcypromine
PGKneo	Neomycin phosphoglycerol kinase; Neomycin glycerol phosphotransferase
Rex1	Reduced expression gene 1 encoding a DNA-binding transcription factor
	known as reduced expression protein 1 or zinc finger protein 42 homolog
rhAT	Recombinant human antithrombin III
rhEPO	Recombinant human erythropoietin
SCNT	Somatic cell nuclear transfer
Sox2	Sex-determining region Y (SRY)-box 2; a member of the high mobility group
	(HMG)-box family of DNA-binding transcription factors
SV-40	Simian virus-40
TALEN	Transcription activator-like effector nuclease
TET3	Ten-eleven translocation 3 protein; TET 5-methylcytosine dioxygenase 3
Xist	X-inactive specific transcript
ZFN	Zinc-finger nuclease
